# A Comprehensive Study to Determine the Residual Elimination Pattern of Major Metabolites of Amoxicillin–Sulbactam Hybrid Molecules in Rats by UPLC–MS/MS

**DOI:** 10.3390/molecules29102169

**Published:** 2024-05-07

**Authors:** Feike Zhao, Xueyan Sun, Jian Li, Junyuan Du, Zhiyi Wu, Shujuan Liu, Liangzhu Chen, Binghu Fang

**Affiliations:** 1 National Laboratory of Safety Evaluation (Environmental Assessment) of Veterinary Drugs, South China Agricultural University, Guangzhou 510642, China; zfk160099@163.com (F.Z.); xueyansun@163.com (X.S.); lijian180506@163.com (J.L.); djy_08093@163.com (J.D.); wuzhiyi202302@163.com (Z.W.); 18739828231@163.com (S.L.); 2 Guangdong Wenshi Dahuanong Biotechnology Co., Ltd., Yunfu 510610, China; che_lizh@163.com

**Keywords:** hybrid molecules, amoxicillin, sulbactam, residue elimination, UPLC–MS/MS technology

## Abstract

Amoxicillin and sulbactam are widely used in animal food compounding. Amoxicillin–sulbactam hybrid molecules are bicester compounds made by linking amoxicillin and sulbactam with methylene groups and have good application prospects. However, the residual elimination pattern of these hybrid molecules in animals needs to be explored. In the present study, the amoxicillin–sulbactam hybrid molecule (AS group) and a mixture of amoxicillin and sulbactam (mixture group) were administered to rats by gavage, and the levels of the major metabolites of amoxicillin, amoxicilloic acid, amoxicillin diketopiperazine, and sulbactam were determined by UPLC–MS/MS. The residue elimination patterns of the major metabolites in the liver, kidney, urine, and feces of rats in the AS group and the mixture group were compared. The results showed that the total amount of amoxicillin, amoxicilloic acid, amoxicillin diketopiperazine, and the highest concentration of sulbactam in the liver and kidney samples of the AS group and the mixture group appeared at 1 h after drug withdrawal. Between 1 h and 12 h post discontinuation, the total amount of amoxicillin, amoxicilloic acid, and amoxicillin diketopiperazine in the two tissues decreased rapidly, and the elimination half-life of the AS group was significantly higher than that in the mixture group (*p* < 0.05); the residual amount of sulbactam also decreased rapidly, and the elimination half-life was not significantly different (*p* > 0.05). In 72 h urine samples, the total excretion rates were 60.61 ± 2.13% and 62.62 ± 1.73% in the AS group and mixture group, respectively. The total excretion rates of fecal samples (at 72 h) for the AS group and mixture group were 9.54 ± 0.26% and 10.60 ± 0.24%, respectively. These results showed that the total quantity of amoxicillin, amoxicilloic acid, and amoxicillin diketopiperazine was eliminated more slowly in the liver and kidney of the AS group than those of the mixture group and that the excretion rate through urine and feces was essentially the same for both groups. The residual elimination pattern of the hybrid molecule in rats determined in this study provides a theoretical basis for the in-depth development and application of hybrid molecules, as well as guidelines for the development of similar drugs.

## 1. Introduction

The misuse of antibiotics has led to a growing problem of drug resistance in pathogenic bacteria [1,2]. On the other hand, high consumption translates into the generation of waste and sewage. Conventional treatment is not able to completely remove many pharmaceutical compounds in water; therefore, this also poses a threat and contributes to drug resistance [3]. Drug resistance in pathogenic bacteria poses a grave challenge to disease prevention and control, as well as a strong threat to human and animal health [4,5]. The long lead time and enormous cost needed for the development of novel drugs make it necessary to use novel technologies to develop novel and improved drugs [6,7]. Drug splicing has been increasingly used as a fast and effective means of developing novel drugs. Drug collocation refers to combining two drugs or two compatible pharmacological groups to form a hybrid molecule. The hybrid molecule exhibits the properties of both drugs/groups to enhance the pharmacological effect of the individual drugs/groups and synergistically complete the therapeutic process and/or reduce the toxic side effects of the individual drugs/groups [8,9]. The collocation of two kinds of drugs is generally implemented using two modes: one mode is the collocation of target enzymes in the animal body by chemical bond hydrolysis to release the original active ingredient, producing a double effect [10,11]; the second mode is collocation by chemical bonding in the target animal body, where the chemical bond is not hydrolyzed by an enzyme and the new hybrid molecule combines different pathogen targets to realize multitarget antimicrobial activity [12,13,14]. It is not difficult to predict the pharmacological activity resulting from the collocation of novel drugs based on the pharmacological effects of the raw materials, providing a basis, and thus shortening the process, for the research and development of novel drugs, thereby saving considerable manpower, material resources, and financial resources [15,16,17].

Amoxicillin (AMO) is a semisynthetic penicillin that is widely used as a broad-spectrum bactericide [18,19]. Like other penicillins, AMO is susceptible to the action of various betalactamases produced by many Gram-positive and Gram-negative microorganisms [20]. As a result, the amoxicillin/sulbactam combination has been commonly used in clinical studies [21,22,23,24]. Combining sulbactam (SBT) with β-lactam antibiotics does not improve the pharmacokinetic profile of SBT in terms of poor oral absorption [25], necessitating the use of novel technologies to develop novel and improved drugs.

**Figure 1:**
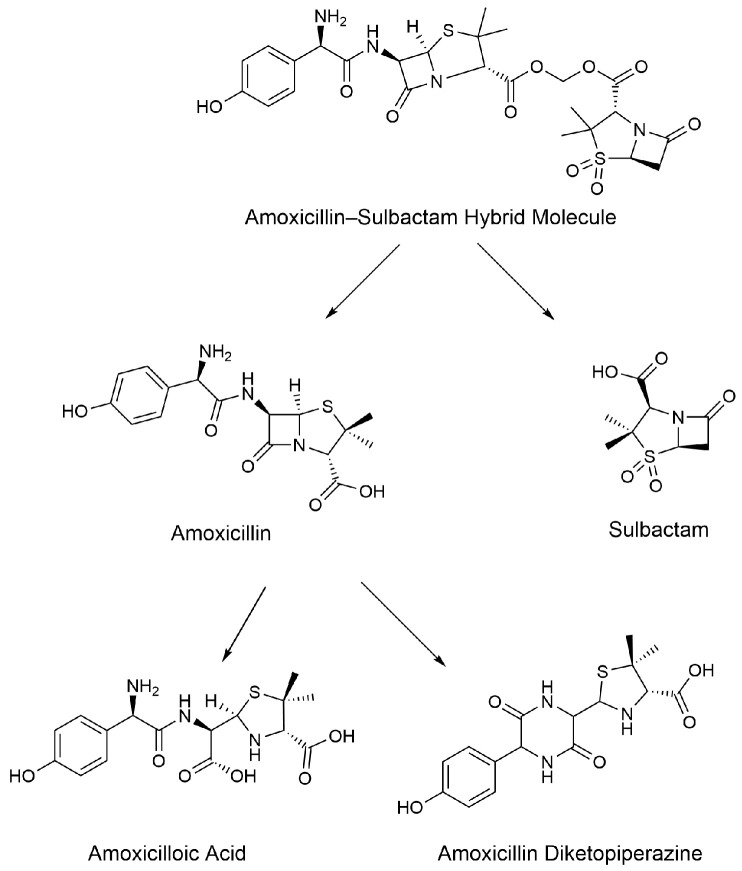
Prediction diagram for the metabolites of the amoxicillin–sulbactam hybrid molecule [28].

We used a synthesis method based on sultazicillin [26,27] to link AMO and SBT through a methylene bridge and synthesize a novel amoxicillin–sulbactam hybrid molecule (AS). In a previous study, after gavage of AS in rats, the heterodimer molecule was found to break the diester bond in rats to produce AMO and SBT at a molar ratio of 1:1. Amoxicillin was further metabolized to amoxicilloic acid (AMA) and amoxicillin diketopiperazine (DIKETO) (Figure 1) [28]. However, the residual elimination pattern of AS in animals is unknown and needs further study.

Several methods have been used for the determination of AMO, AMA, DIKETO, and SBT, including the use of a UPLC–photodiode array detector (PAD) [29], a high-performance liquid chromatography (HPLC)–ultraviolet detector (UV) [30,31,32,33], an HPLC–fluorescence detector (FLD) [34], and reversed-phase (RP)-HPLC-FLD [35], as well as LC–MS/MS [36,37,38], HPLC–ESI/MS/MS [39,40], and UPLC–MS/MS [41,42] analyses.

Administered AMO is metabolized in large quantities in the body to amoxicilloic acid, DIKETO, and AMO, and its metabolites are widely distributed in the fluids and tissues of the animal body, becoming most concentrated in the liver and kidneys [43]. Administered SBT is widely distributed in the fluids and tissues of the animal body, becoming most concentrated in the liver, kidneys, and lungs and existing as a prodrug [44]. In clinical practice, AMO and SBT are generally used in combination [21]. The rate of absorption and elimination of the AMO and SBT combination in animals is faster than those of the individual drugs; the combination drug is mainly eliminated from the body through urine but can partially be eliminated through feces [45,46,47]. Most studies on the excretion pattern of AMO and SBT have only considered urinary excretion, and fewer studies have been performed on fecal excretion. Therefore, the excretion of AMO and SBT through the urine and feces of animals needs further study.

The residual elimination pattern of AS in animals is still unclear and requires further study. Therefore, the present study was conducted to develop and validate a UPLC-MS/MS method for the quantification of AMO, AMA, DIKETO, and SBT, the major metabolites of AS, in the liver, kidney, urine, and feces of rats. Furthermore, this study investigated the changes in the major metabolites of AS in the liver, kidney, urine, and feces following the administration of AS and an amoxicillin/sulbactam mixture to rats. This study elucidated the residual elimination pattern of AS in animals and provided a theoretical basis for the clinical application of AS.

## 2. Results

### 2.1. Method Validation

#### 2.1.1. Specificity

**Figure 2:**
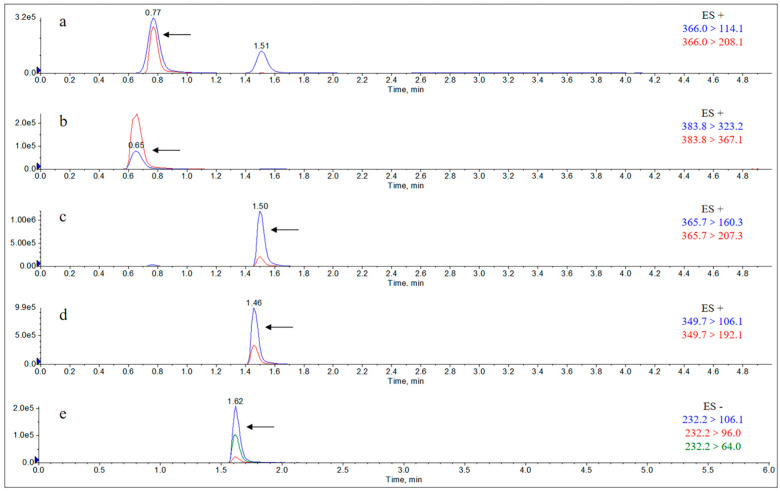
Extract Ion Chromatograms (EICs) of standard solutions of each metabolite and the internal standard of AS (10 μg/mL). Note: (**a**) amoxicillin; (**b**) amoxicilloic acid; (**c**) amoxicillin diketopiperazine; (**d**) ampicillin; and (**e**) sulbactam.

**Figure A1:**
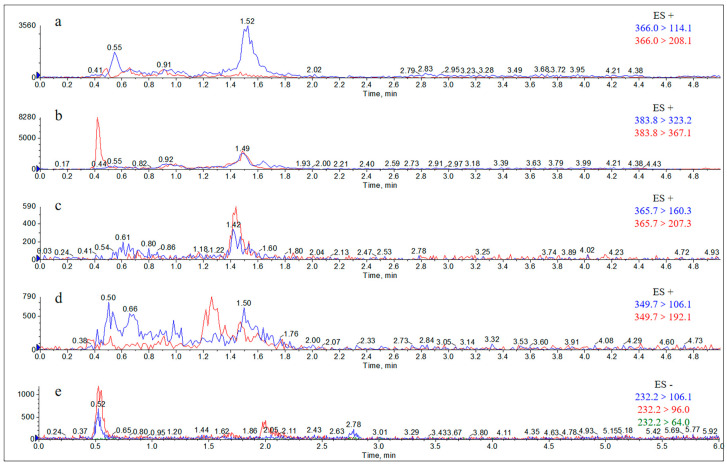
Extract Ion Chromatograms (EICs) of the rat liver blank. Note: (**a**) amoxicillin; (**b**) amoxicilloic acid; (**c**) amoxicillin diketopiperazine; (**d**) ampicillin; and (**e**) sulbactam.

**Figure A2:**
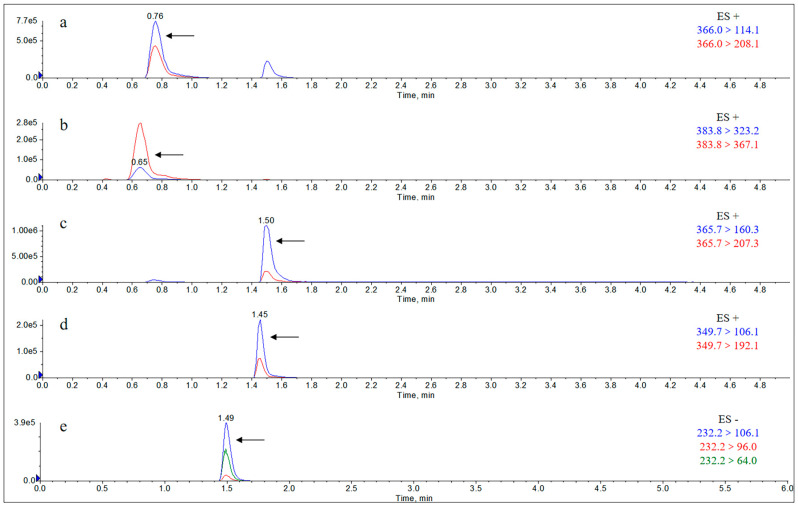
Extract Ion Chromatograms (EICs) of the rat liver matrix addition standard solution (10 μg/g). Note: (**a**) amoxicillin; (**b**) amoxicilloic acid; (**c**) amoxicillin diketopiperazine; (**d**) ampicillin; and (**e**) sulbactam.

**Figure A3:**
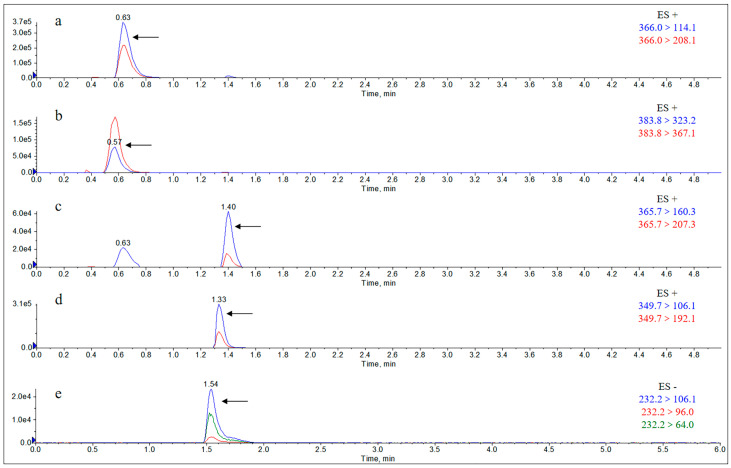
Extract Ion Chromatograms (EICs) of individual metabolites in the rat liver 0.5 h after gavage of AS. Note: (**a**) amoxicillin; (**b**) amoxicilloic acid; (**c**) amoxicillin diketopiperazine; (**d**) ampicillin; and (**e**) sulbactam.

**Figure A4:**
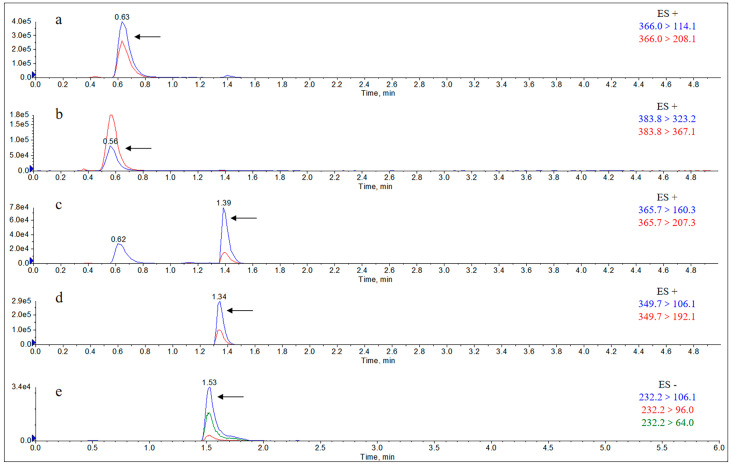
Extract Ion Chromatograms (EICs) of individual metabolites in the rat liver 0.5 h after gavage of the drug mixture. Note: (**a**) amoxicillin; (**b**) amoxicilloic acid; (**c**) amoxicillin diketopiperazine; (**d**) ampicillin; and (**e**) sulbactam.

**Figure A5:**
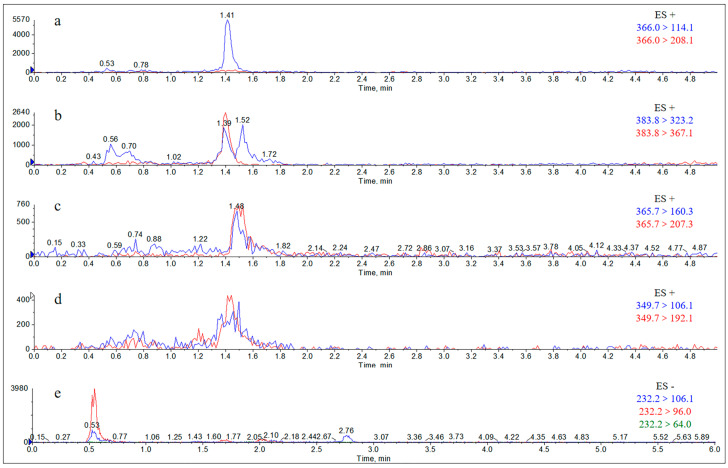
Extract Ion Chromatograms (EICs) of blank rat kidney. Note: (**a**) amoxicillin; (**b**) amoxicilloic acid; (**c**) amoxicillin diketopiperazine; (**d**) ampicillin; (**e**) sulbactam.

**Figure A6:**
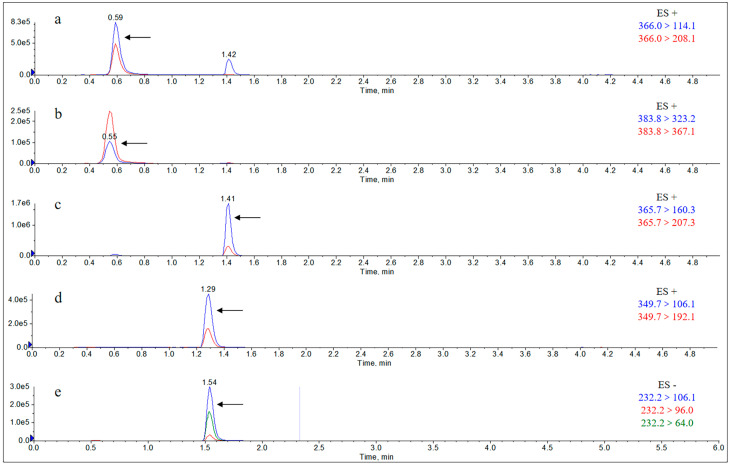
Extract Ion Chromatograms (EICs) of rat kidney matrix addition standard solution (10 μg/g). Note: (**a**) amoxicillin; (**b**) amoxicilloic acid; (**c**) amoxicillin diketopiperazine; (**d**) ampicillin; (**e**) sulbactam.

**Figure A7:**
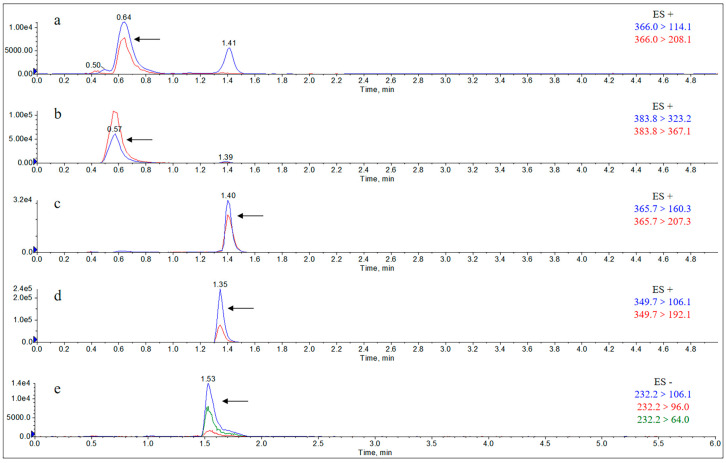
Extract Ion Chromatograms (EICs) of individual metabolites in the kidney of rats 0.5 h after gavage of AS. Note: (**a**) amoxicillin; (**b**) amoxicilloic acid; (**c**) amoxicillin diketopiperazine; (**d**) ampicillin; (**e**) sulbactam.

**Figure A8:**
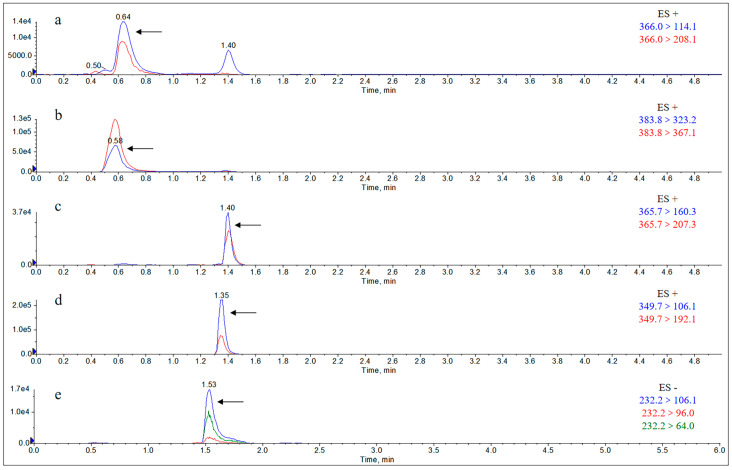
Extract Ion Chromatograms (EICs) of individual metabolites in the kidney of rats 0.5 h after gavage of the mixture. Note: (**a**) amoxicillin; (**b**) amoxicilloic acid; (**c**) amoxicillin diketopiperazine; (**d**) ampicillin; (**e**) sulbactam.

**Figure A9:**
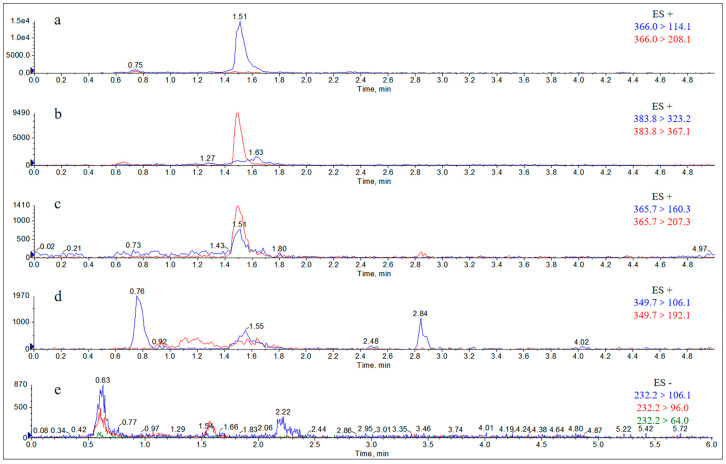
Extract Ion Chromatograms (EICs) of blank rat urine. Note: (**a**) amoxicillin; (**b**) amoxicilloic acid; (**c**) amoxicillin diketopiperazine; (**d**) ampicillin; (**e**) sulbactam.

**Figure A10:**
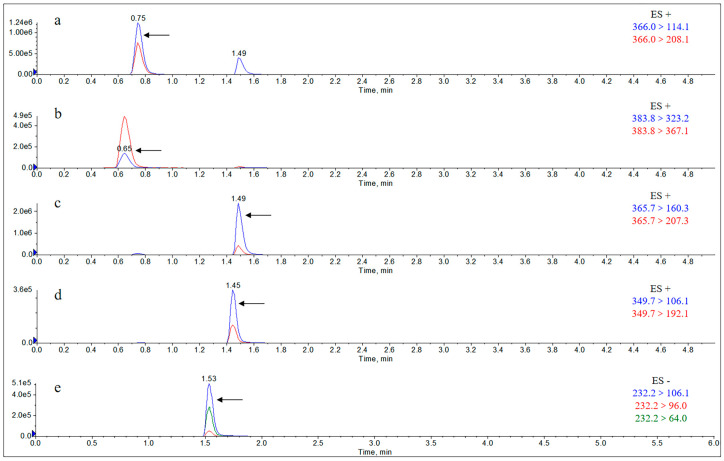
Extract Ion Chromatograms (EICs) of rat urine matrix addition standard solution (10 μg/mL). Note: (**a**) amoxicillin; (**b**) amoxicilloic acid; (**c**) amoxicillin diketopiperazine; (**d**) ampicillin; (**e**) sulbactam.

**Figure A11:**
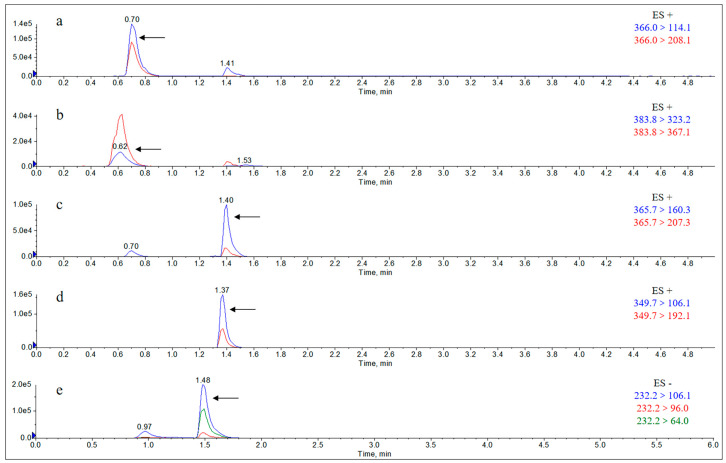
Extract Ion Chromatograms (EICs) of individual metabolites in the urine of rats 12 h after gavage of AS. Note: (**a**) amoxicillin; (**b**) amoxicilloic acid; (**c**) amoxicillin diketopiperazine; (**d**) ampicillin; (**e**) sulbactam.

**Figure A12:**
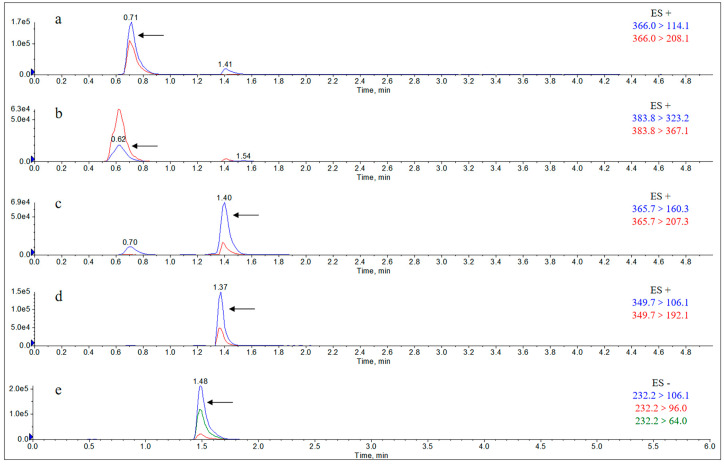
Extract Ion Chromatograms (EICs) of individual metabolites in the urine of rats 12 h after gavage of the mixture. Note: (**a**) amoxicillin; (**b**) amoxicilloic acid; (**c**) amoxicillin diketopiperazine; (**d**) ampicillin; (**e**) sulbactam.

**Figure A13:**
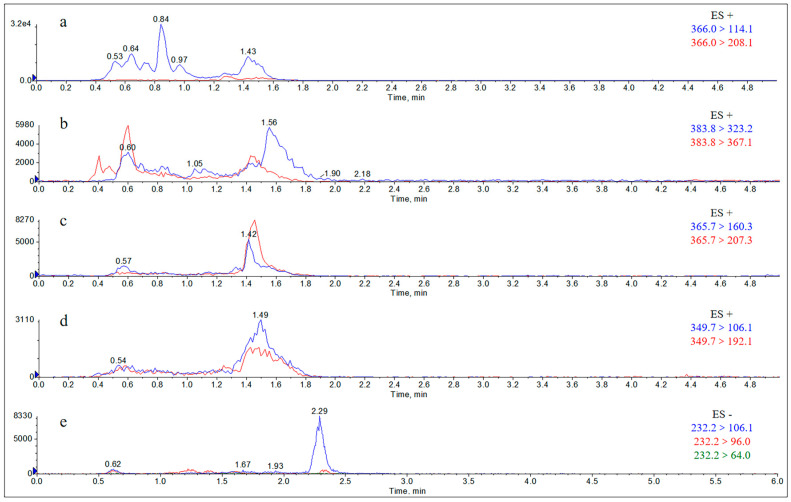
Extract Ion Chromatograms (EICs) of blank rat feces. Note: (**a**) amoxicillin; (**b**) amoxicilloic acid; (**c**) amoxicillin diketopiperazine; (**d**) ampicillin; (**e**) sulbactam.

**Figure A14:**
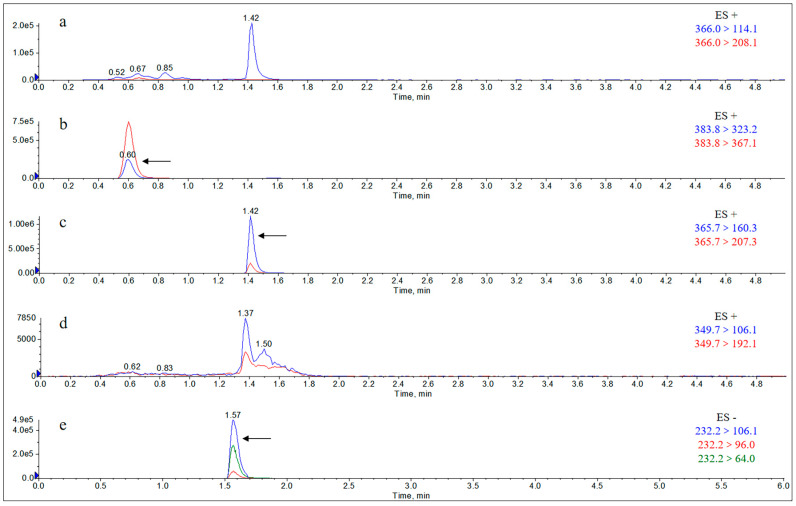
Extract Ion Chromatograms (EICs) of rat feces matrix addition standard solution (10 μg/g). Note: (**a**) amoxicillin; (**b**) amoxicilloic acid; (**c**) amoxicillin diketopiperazine; (**d**) ampicillin; (**e**) sulbactam.

**Figure A15:**
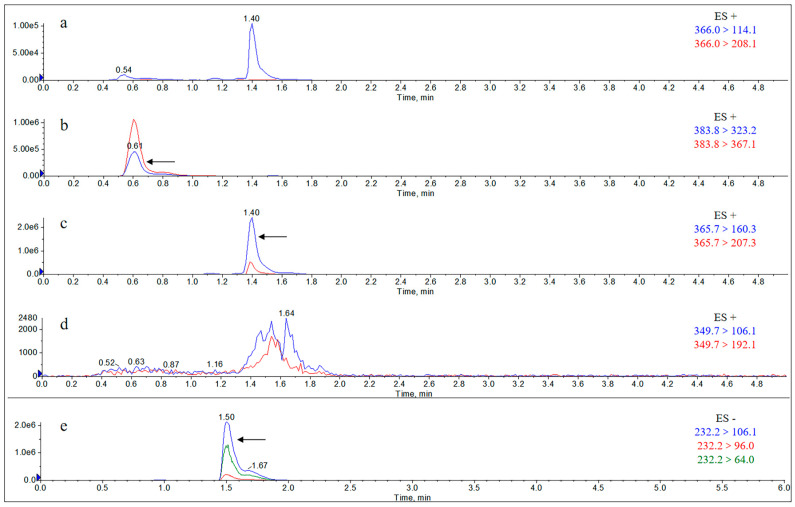
Extract Ion Chromatograms (EICs) of individual metabolites in the feces of rats 12 h after gavage of AS. Note: (**a**) amoxicillin; (**b**) amoxicilloic acid; (**c**) amoxicillin diketopiperazine; (**d**) ampicillin; (**e**) sulbactam.

**Figure A16:**
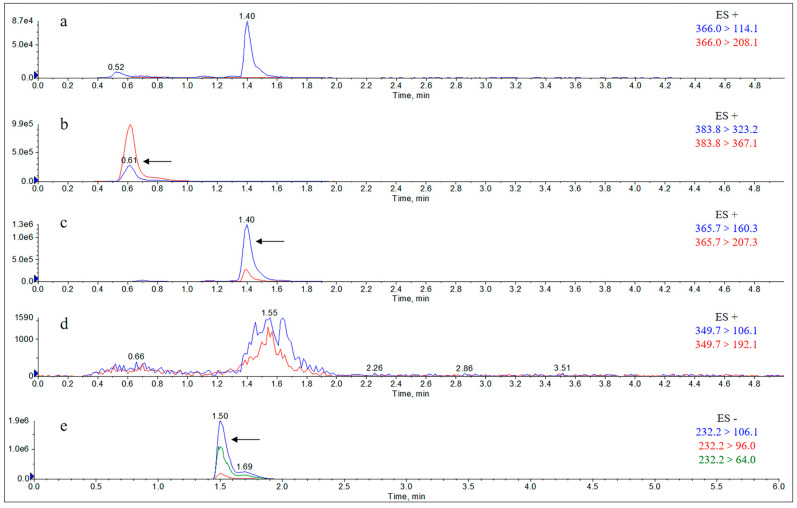
Extract Ion Chromatograms (EICs) of individual metabolites in the feces of rats 12 h after gavage of the mixture. Note: (**a**) amoxicillin; (**b**) amoxicilloic acid; (**c**) amoxicillin diketopiperazine; (**d**) ampicillin; (**e**) sulbactam.

The Extract Ion Chromatograms (EICs) of the standard solutions of each metabolite and the internal standard of AS are shown in Figure 2. The Extract Ion Chromatograms (EICs) of the blank samples, blank samples with added analytes, and actual collection samples from the liver are shown in Figure A1, Figure A2, Figure A3 and Figure A4, and the Extract Ion Chromatograms (EICs) of the blank samples, blank samples with added analytes, and actual collection samples from the kidney, urine, and feces are shown in Figure A5, Figure A6, Figure A7, Figure A8, Figure A9, Figure A10, Figure A11, Figure A12, Figure A13, Figure A14, Figure A15 and Figure A16. The results showed that the Extract Ion Chromatogram (EIC) retention times for AMO, AMA, DIKETO, SBT, and Ampicillin (AMP) were approximately 0.77 min, 0.65 min, 1.50 min, 1.62 min, and 1.46 min, respectively.

#### 2.1.2. Limits of Detection (LODs) and Quantitation (LOQs)

The limits of detection and quantification were determined for the four test analytes, AMO, AMA, DIKETO, and SBT. The concentration of the added mass at a signal-to-noise ratio SN ≥ 3 was determined as the lowest limit of detection (LOD), and the concentration of the added mass at S/N ≥ 10 was determined as the limit of quantification (LOQ); the results are shown in Table 1.

#### 2.1.3. Linearity

The linear regression equations, coefficients of determination, and linear ranges of the major metabolites of AS in the rat samples are shown in the attached Table A1, Table A2, Table A3 and Table A4. The peak area ratios (x) of the quantitative transition of AMO, AMA, and DIKETO to the quantitative transition of the internal standard, AMP, for each of the test standards in the blank rat liver, kidney, and urine samples were linearly correlated with the corresponding concentration ratios (y) over the specified concentration ranges with good linearity. For the blank rat fecal samples of each test standard in the specified concentration range, the concentrations (c) of AMA and DIKETO were linearly correlated with the peak area (s), with good linearity. In the blank rat liver, kidney, urine, and feces samples of each test standard, the concentration (c) of SBT was linearly correlated with the peak area (s) within the specified concentration range, with good linearity.

#### 2.1.4. Recovery and Precision

The recovery and precision results are shown in Table A5, Table A6, Table A7 and Table A8. The recoveries and precision were determined for the four analytes in four rat samples at low, medium, and high spiked levels for samples pretreated according to “Section 4.2.1”. The recoveries ranged from 71.59% to 110.92%, the intra-batch variability ranged from 1.37% to 11.44%, and the inter-batch variability ranged from 3.53% to 10.53%.

### 2.2. Residual Elimination Results

**Figure 3:**
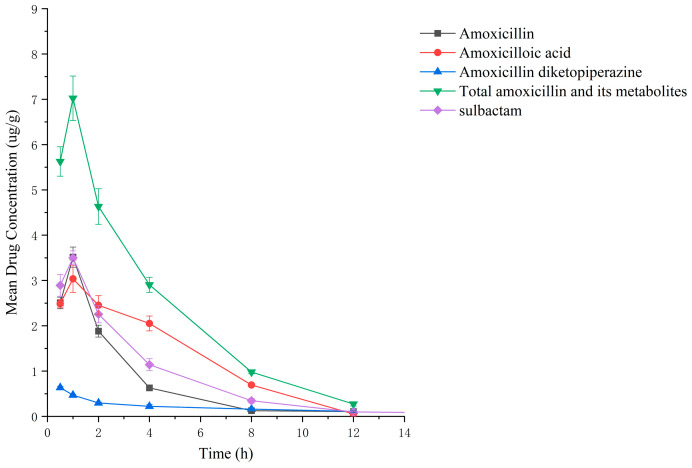
Drug concentration–time profiles in the liver of rats after administration of AS.

**Figure 4:**
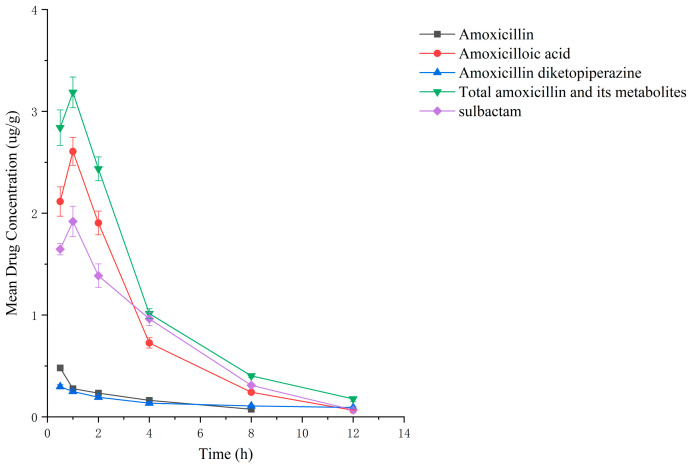
Drug concentration–time profiles in the kidney of rats after administration of AS.

**Figure 5:**
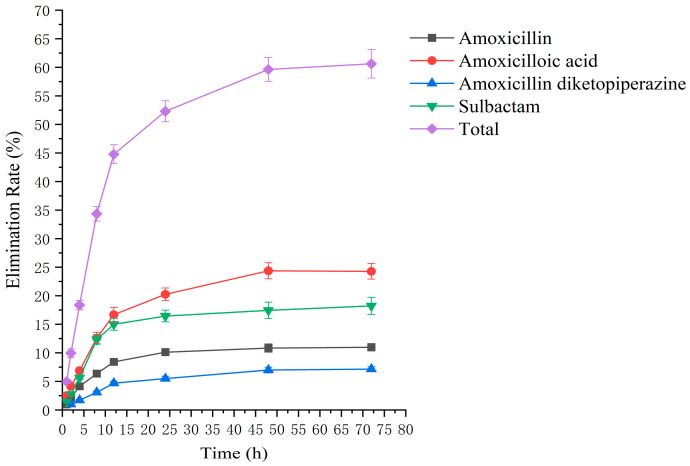
The rate of drug excretion through urine after administration of AS in rats.

**Figure 6:**
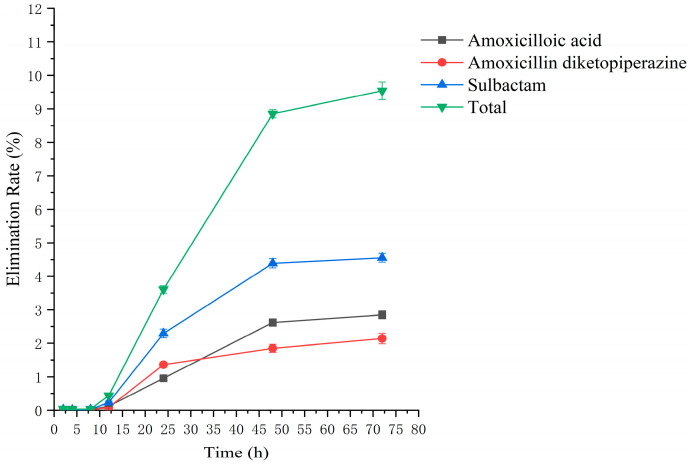
The rate of drug excretion through feces after administration of AS in rats.

**Figure 7:**
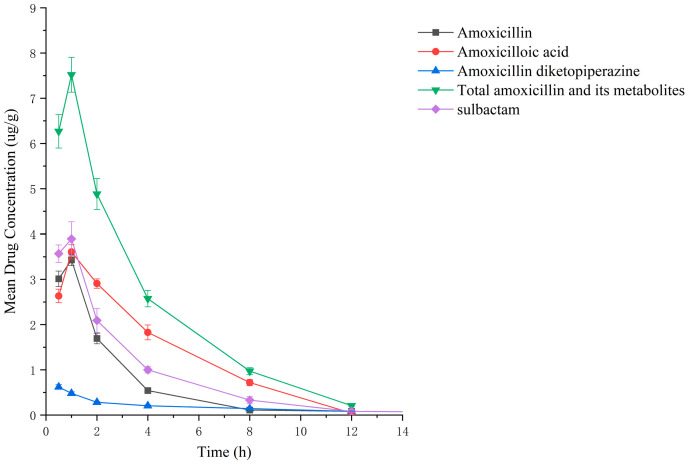
Drug concentration–time profiles in the liver of rats after administration of amoxicillin/sulbactam mixture.

**Figure 8:**
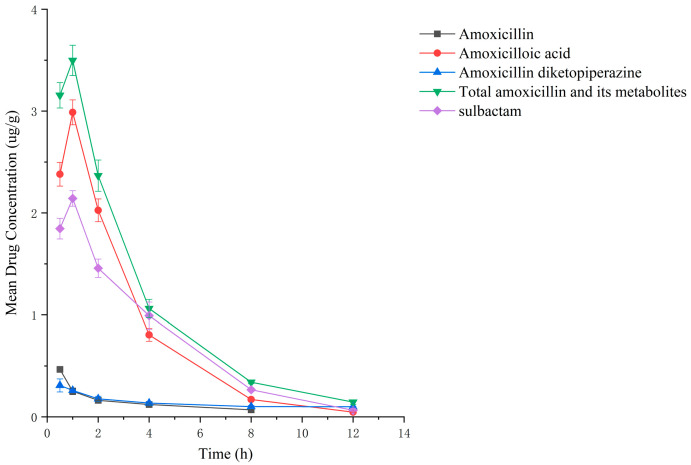
Drug concentration–time profiles in the kidney of rats after administration of amoxicillin/sulbactam mixture.

**Figure 9:**
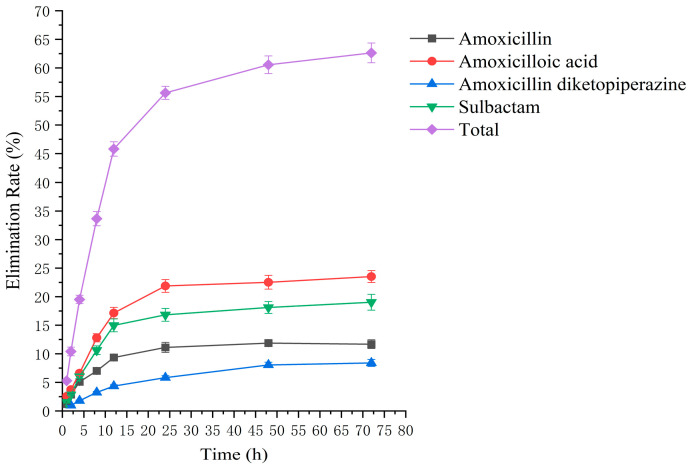
The rate of drug excretion through urine after administration of amoxicillin/sulbactam mixture in rats.

**Figure 10:**
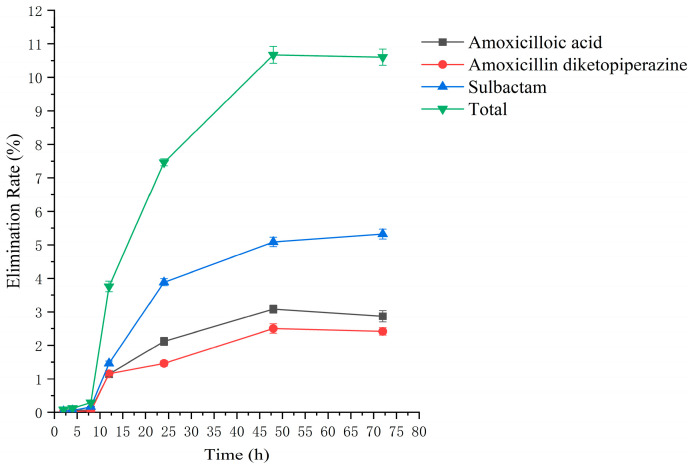
The rate of drug excretion through feces after administration of amoxicillin/sulbactam mixture in rats.

Drug administration and sample treatment were carried out according to the test protocol, and the samples were detected by HPLC tandem mass spectrometry analysis. Table A9, Table A10, Table A11, Table A12, Table A13, Table A14, Table A15 and Table A16 show the drug concentrations in the liver and kidney at each sampling time point, as well as the urinary and fecal excretion rates, after the administration of AS (AS group) and the amoxicillin/sulbactam mixture (mixture group) by gavage. Figure 3, Figure 4, Figure 5, Figure 6, Figure 7, Figure 8, Figure 9 and Figure 10 show the drug concentration–time profiles in the liver and kidney, as well as the urinary and fecal excretion rates. As shown in Figure 3, Figure 4, Figure 5, Figure 6, Figure 7, Figure 8, Figure 9 and Figure 10, the total concentrations of AMO, AMA, and DIKETO and the SBT concentration in the liver and kidney of the AS group and the mixture group increased gradually from 0.5 h to 1 h and then declined rapidly from 1 h to 12 h. For the major metabolites in the AS and mixture groups, the total urinary excretion rate was 60.61 ± 2.13% and 62.62 ± 1.73%, respectively, and the total excretion rate via feces was 9.54 ± 0.26% and 10.60 ± 0.24%, respectively. AMO and SBT were rapidly absorbed internally with a shorter time to peak and rapidly eliminated by the organism. Table 2 shows the elimination equations and half-lives of the major metabolites in the rat liver and kidney. As shown in Table 2, the total amount of AMO and its metabolites in the AS group was significantly higher than that in the mixture group (*p* < 0.05), and there was no significant difference in the elimination half-life of SBT (*p* > 0.05).

## 3. Discussion

In this study, ESI (+) was used to detect AMO, AMA, and DIKETO in the liver, kidney, and urine using ampicillin as an internal standard. Due to the heterogeneity of the fecal matrix and the rapid degradation of β-lactam antibiotics in feces [48], the external standard method was used to detect amoxicilloic acid and DIKETO in feces. SBT exhibited a strong response under ESI (−); therefore, ESI (−) was used to detect SBT in the liver, kidney, and feces by the external standard method. The method validation results showed that the LODs of all analytes in the liver, kidney, urine, and feces were lower than 0.025 μg/mL or μg/g, and the LOQs were lower than 0.05 μg/mL or μg/g. The recovery rate of AMO was between 91.55 and 111.46%, the recovery rate of amoxicilloic acid was between 80.56 and 110.62%, the recovery rate of DIKETO was between 80.07 and 110.34%, and the recovery rate of SBT was between 71.59 and 98.24%. Therefore, the extraction of AMO, AMA, and DIKETO with water as an extractant and the extraction of SBT with ethyl acetate as an extractant was found to be more effective. The sample treatment method used in this study showed high recovery and sensitive detection compared to the methods reported in the literature [49,50,51].

After the administration of the amoxicillin–sulbactam hybrid molecule (AS group) and the amoxicillin/sulbactam mixture (mixture group) by gavage, the total concentrations of AMO, AMA, and DIKETO in the liver and kidney and the SBT concentration increased gradually from 0.5 h to 1 h and then decreased rapidly from 1 h to 12 h. AMO and SBT were rapidly absorbed internally with a shorter time to peak and rapidly eliminated by the organism, consistent with results reported in the literature [52,53].

Between 0.5 h and 1 h after drug administration, the concentrations of AMO and AMA in the liver gradually increased and were similar in value; between 1 h and 12 h after drug administration, the AMA concentration decreased more slowly than the AMO concentration, indicating that large quantities of AMO were metabolized to AMA in the liver. In the kidney, the concentration of AMA was higher and the concentrations of AMO and AMA were lower than in the liver; the concentrations of AMO and AMA gradually decreased after 0.5 h, whereas the AMA concentration gradually increased from 0.5 h to 1 h and then decreased rapidly from 1 h to 12 h. For the liver and kidney samples of rats in the AS and mixture groups, the total quantity of AMO, AMA, and DIKETO appeared together with the highest residual quantity of SBT at approximately 1 h post discontinuation, where the distribution of each drug followed the rule liver > kidney. Between 1 h post discontinuation and 12 h post discontinuation, the total quantity of AMO, AMA, and DIKETO and the residual quantity of SBT decreased rapidly in both tissues. The elimination half-life (t_1/2β_) parameters of the AS group and the mixture group were analyzed by a *t*-test using SPSS 24.0 software, and the results showed that the differences in the t_1/2β_ of the two groups were statistically significant for the total quantity of AMO, AMA, and DIKETO in the liver and kidney (*p* < 0.05); statistically significant for AMO in the liver (*p* < 0.05); statistically significant for AMA in the liver (*p* < 0.05); highly statistically significant for AMA in the kidney (*p* < 0.01); and not statistically significant for SBT in the liver and kidney. These results indicate that the total quantity of AMO, AMA, and DIKETO was eliminated more slowly in the liver and kidney of rats in the AS group than in the mixture group.

The excretion rates of the four substances through urine and feces were basically the same in the AS and mixture groups, with total excretion rates of 60.61 ± 2.13% and 62.62 ± 1.73%, respectively, through urine and 9.54 ± 0.26% and 10.60 ± 0.24%, respectively, through feces. The excretion rates of AMO, AMA, and DIKETO in urine from the AS and mixture groups were 10.98 ± 0.65% and 11.68 ± 0.76%, 24.26 ± 1.36% and 23.52 ± 1.04%, and 7.15 ± 0.42% and 8.40 ± 0.62%, respectively, which shows that AMO was excreted in urine mainly as its metabolite AMA. The total excretion rate through urine for both groups was similar to that reported in the literature [54,55].

## 4. Materials and Methods

### 4.1. Materials

#### 4.1.1. Chemicals and Reagents

The following chemicals and reagents were used in this study:

Amoxicillin (87.00%, Lot No. 130409-201913) was obtained from the China National Institute for Food and Drug Control (Beijing, China);

Sulbactam (98.54%, Lot No. DM21022603) was obtained from Guangzhou Juanmu Biotechnology Co., Ltd. (Guangzhou, China);

Amoxicillin diketone piperazine (97.24%, Lot No. DM20051896) was obtained from Guangzhou Juanmu Biotechnology Co., Ltd. (Guangzhou, China);

Amoxicilloic acid (97.87%, Lot No. A634265) was obtained from Guangdong Boyan Scientific Instrument Co., Ltd. (Zhaoqing, China);

Ampicillin (98.00%, Lot No. A830931) used as an internal standard was obtained from Shanghai Maclin Biochemical Technology Co., Ltd. (Shanghai, China);

AS (content detected by HPLC: 96.50%; amoxicillin content: 55.50%; sulbactam content: 35.37%), the NMR spectrum of AS is: 1H NMR (600 MHz, DMSO-*d*_6_) δ 9.82 (s, 1H, OH), 9.28 (d, *J* = 7.5 Hz, 1H, NH), 8.57 (s, 1H, NH), 7.28 (d, *J* = 8.6 Hz, 2H), 6.80 (d, *J* = 8.6 Hz, 2H), 5.91 (q, *J* = 6.1 Hz, 2H, OCH2O), 5.59 (t, *J* = 4.8 Hz, 1H), 5.45 (d, *J* = 4.1 Hz, 1H), 5.20 (dd, *J* = 4.6, 1.8 Hz, 1H), 4.95 (s, 1H), 4.56 (s, 1H), 4.42 (s, 1H), 4.03 (q, *J* = 7.1 Hz, 1H), 3.73–3.65 (m, 1H), 1.49 (s, 3H), 1.46 (s, 3H), 1.36 (s, 3H), 1.35 (s, 3H);

Acetonitrile, methanol, and formic acid were obtained at chromatographic grade from Thermo Fisher Scientific (China) Co., Ltd. (Shanghai, China).

#### 4.1.2. Instruments

The following equipment was used in this study:

An ultra-high-performance liquid chromatograph: an Agilent 1290 Infinity II ultra-high-performance liquid chromatography system, equipped with quaternary pump, degassing pump, automatic sampler, and column oven (Agilent Technologies, Ltd., Beijing, China);

An electrospray tandem triple quadrupole mass spectrometer: a Triple QuadTM4500 liquid mass spectrometer, AB SCIEX company, equipped with Analyst 1.6.3 software (Agilent Technologies, Ltd., Beijing, China);

A rat metabolic cage: SA106 (Guangzhou Kaige Biotechnology Co., Ltd., Guangzhou, China);

A SAX solid-phase extraction column: 60 mg/3 mL and 50pk-00513-11009 (Yuexu Technology Co., Ltd., Shanghai, China).

#### 4.1.3. Solution Preparation

The following solutions were prepared using the procedures described.

A total of 1.00 mg/mL amoxicillin standard solution: 11.49 mg of an amoxicillin standard was weighed into a 10 mL brown bottle and dissolved in 50% (*v*/*v*) acetonitrile in water to make the volume 10 mL. The solution was stored at −80 °C until use.

A total of 1.00 mg/mL amoxicillin acid standard solution: 10.22 mg of an amoxicillin acid standard was weighed into a 10 mL brown bottle and dissolved in ultrapure water to make the volume 10 mL. The solution was stored at −80 °C until use.

A total of 1.00 mg/mL amoxicillin diketopiperazine standard solution: 10.28 mg of an amoxicillin diketopiperazine standard was weighed into a 10 mL brown bottle and dissolved in 50% (*v*/*v*) acetonitrile in water to make the volume 10 mL. The solution was stored at −80 °C until use.

A total of 1.00 mg/mL sulbactam standard solution: 10.20 mg of a sulbactam standard was weighed into a 10 mL brown bottle and dissolved in ultrapure water to make the volume 10 mL. The solution was stored at −80 °C until use.

A total of 1 mol/L hydrochloric acid solution: 25 mL of 36–38% concentrated hydrochloric acid was measured. Then, 275 mL of water was added to the acid, and the solution was mixed and reserved for use.

A total of 5 mol/L sodium hydroxide: 20 g of sodium hydroxide was accurately weighed and dissolved in 100 mL of water.

A total of 0.1 mol/L phosphate buffer: 13.6 g of potassium dihydrogen phosphate was accurately weighed and mixed with ultrapure water to make the volume 1 L. Then, 5 mol/L of sodium hydroxide was added to the solution to adjust the pH to 8.0.

Elution Solution A: 50 mL of methanol and 10 mL of formic acid were added to 450 mL of pure water, and the resulting solution was mixed well.

Elution Solution B: 2 mL of formic acid was added to 100 mL of methanol, and the resulting solution was mixed well.

### 4.2. Detection Methods for Major Metabolites of AS

#### 4.2.1. UPLC–MS/MS for Major Metabolites of AS

##### UPLC–MS/MS Instrumental Conditions for AMO, AMA, DIKETO, and AMP

Liquid chromatography conditions include the following:

Column: Waters ACQUITY UPLC BEH C18 (2.1 mm × 50 mm, 1.7 μm) (Waters Corporation, USA) (Beijing, China); mobile phase: 0.1% formic acid in acetonitrile and 0.1% formic acid in water; flow rate: 0.35 mL/min; column temperature: 25 °C; and injection volume: 5.0 μL. The procedure of gradient elution of the mobile phase is shown in Table 3.

Mass spectrometry conditions: The mass spectrometry parameters were optimized using electrospray ionization source (ESI), positive ion scanning, and multiple reaction monitoring (MRM) modes. The optimal parameters were as follows: electrospray voltage (IS): 5500 V; nebulizing gas pressure (GS1): 50 psi; auxiliary gas flow rate (GS2): 50 L/min; curtain gas pressure (CUR): 40 psi; ion source temperature (TEM): 550 °C; and collision chamber pressure (CAD): 9 psi. The decluster voltage (DP) and collision energy (CE) of amoxicillin, amoxicilloic acid, amoxicillin diketopiperazine, and ampicillin, are shown in Table 4.

The pretreatment of samples containing AMO, AMA, and DIKETO is as follows.

Liver and kidney samples: 0.5 g of a sample was accurately weighed into a 50 mL centrifuge tube. A 10 μL volume of a 50 μg/mL ampicillin internal standard was added to the tube, and the solution was mixed well by vortexing. A volume of 2 mL of water and 2 mL of acetonitrile were added to the solution, which was vortexed thoroughly for 1 min. Then, an additional 2 mL of acetonitrile was added to the solution, which was vortexed thoroughly for 1 min, ultrasonically extracted for 30 min, and centrifuged at 3780 rcf for 5 min. The supernatant was transferred to a 50 mL centrifuge tube. The precipitate was mixed with 1 mL of water and 4 mL of acetonitrile, and the mixture was vortexed thoroughly for 1 min, sonicated for 30 min, and centrifuged at 3780 rcf for 5 min. Then, the supernatants were combined. The precipitate was vortexed with 5 mL of water for 1 min, sonicated for 30 min, and centrifuged at 11,140 rcf for 5 min. The supernatants were combined.

A volume of 9 mL of dichloromethane was added to the combined extract, and the mixture was vortexed for 1 min. The mixture was centrifuged at 3780 rcf for 5 min. Then, 3 mL of the supernatant was aspirated into a 15 mL centrifuge tube and 5 mL of n-hexane was added to the tube, and the mixture was vortexed for 1 min. The mixture was centrifuged at 3780 rcf for 5 min, and the upper layer of n-hexane was discarded. The supernatant was filtered through a 0.22 μm microporous membrane and subjected to UPLC–MS/MS analysis.

Urine: 100 μL of a sample was accurately pipetted into a 2 mL centrifuge tube. A 10 μL volume of a 10 μg/mL ampicillin internal standard was added to the tube. The mixture was vortexed, and 1 mL of a 0.1 mol/L phosphate buffer was added to the tube. The mixture vortexed for 1 min. The aforementioned liquids were loaded into a SAX solid-phase extraction (SPE) column activated with 3 mL of methanol, 3 mL of water, and 3 mL of a 0.1 mol/L phosphate buffer. The flow rate was controlled to within 3 mL/min. The flow was terminated, and the columns were washed with 3 mL of a phosphate buffer. Finally, sequential elution was performed using 3 mL of Eluent A and 1 mL of Eluent B. The eluent was collected and filtered through a 0.22 μm microporous membrane for use in UPLC–MS/MS analysis.

Feces: 0.5 g of a sample was accurately weighed into a 50 mL centrifuge tube, and a 10 mL volume of water was added to the tube. The mixture was vortexed thoroughly for 1 min, ultrasonically extracted for 30 min, and centrifuged at 7740 rcf for 5 min. The supernatant was transferred to a 50 mL centrifuge tube. A 5 mL volume of water was added to the tube. The mixture was vortexed for 1 min, sonicated for 30 min, and centrifuged at 11,140 rcf for 5 min. The supernatants were combined.

A 9 mL volume of dichloromethane was added to the combined supernatant. The mixture was vortexed thoroughly for 1 min and centrifuged at 3780 rcf for 5 min. The supernatants were aspirated, passed through a 0.22 μm microporous filter membrane, and subjected to UPLC–MS/MS analysis.

##### UPLC–MS/MS Instrumental Conditions for SBT

The liquid chromatography conditions are as follows.

Chromatographic column: Waters ACQUITY UPLC BEH C18 (2.1 mm × 50 mm, 1.7 μm) (Waters Corporation, USA) (Beijing, China); mobile phase: 0.1% formic acid in acetonitrile for Phase A and 0.1% formic acid in water for Phase B; flow rate: 0.30 mL/min; column temperature: 25 °C; and injection volume: 10.0 μL. The gradient elution procedure for the mobile phase is shown in Table 5.

Mass spectrometry conditions: The mass spectrometry parameters were optimized using electrospray ionization source (ESI), negative ion scanning, and multiple reaction monitoring (MRM) modes. The optimal parameters were as follows: electrospray voltage (IS): 5500 V; nebulizing gas pressure (GS1): 50 psi; auxiliary gas flow rate (GS2): 50 L/min; curtain gas pressure (CUR): 40 psi; ion source temperature (TEM): 550 °C; and collision chamber pressure (CAD): 9 psi. The decluster voltage (DP) and collision energy (CE) of sulbactam are shown in Table 6.

The pretreatment of SBT in the samples is as follows.

Liver, kidney, and feces: 0.5 g of a sample was placed in a 50 mL centrifuge tube. A 1 mL volume of 1 mol/L dilute hydrochloric acid was added to the tube, and the mixture was vortexed thoroughly for 1 min. Then, 5 mL of ethyl acetate was added to the tube. The mixture was vortexed thoroughly for 1 min, stirred for 30 min, and centrifuged at 3780 rcf for 5 min. The supernatant was transferred to a 15 mL centrifuge tube. The extraction process was repeated, and the supernatants were combined. The product was blown dry under nitrogen at 40 °C. The residue was redissolved in 5 mL of 10% acetonitrile in water and then centrifuged at 7740 rcf for 5 min. The supernatant was filtered through a 0.22 μm filter membrane and subjected to UPLC–MS/MS analysis.

Urine: A 100 μL volume of the sample was aspirated into a 5 mL centrifuge tube, followed by adding 100 μL of 1 mol/L dilute hydrochloric acid to the tube. The mixture was vortexed thoroughly for 1 min, and 2 mL of ethyl acetate was added to the mixture. Then, the mixture was vortexed thoroughly for 1 min, stirred for 10 min, and centrifuged at 1240 rcf for 3 min. The supernatant was transferred into a 5 mL centrifuge tube and extracted repeatedly. The supernatants were combined and blown dry under nitrogen gas at 40 °C. The residue was redissolved in 0.5 mL of 10% acetonitrile in water, and the supernatant was passed through a 0.22 μm filter membrane and subjected to UPLC–MS/MS analysis.

#### 4.2.2. Limits of Detection (LODs) and Quantitation (LOQs)

The LODs and LOQs of the method were assessed using rat blank liver, kidney, urine, and feces samples spiked with a standard. The standard solutions of the four substances to be assayed were spiked at low concentrations of 0.0025, 0.01, 0.025, and 0.05 μg/mL or μg/g. The samples were then analyzed by HPLC–MS/MS, with five replicates at each concentration. The limit of quantification (LOQ) was set at the spike concentration for which the signal-to-noise ratio of the daughter ions was greater than or equal to 10 (S/N > 10); the limit of detection (LOD) was set at the spike concentration at which the signal-to-noise ratio of the daughter ions greater than or equal to 3 (S/N > 3).

#### 4.2.3. Linearity

The linearity of the method was assessed by analyzing blank samples of uncontaminated rat samples (liver, kidney, urine, and feces) with different AMO, AMA, DIKETO, and SBT spiked concentrations to establish a calibration curve. AMO, AMA, DIKETO, and SBT standard solutions were diluted and mixed in 0.025, 0.1, 0.5, 1, 2.5, 5, 7.5, and 10 μg/g or μg/mL (no AMO was added to the calibration curves for AMA and DIKETO in feces), and each tissue was repeated three times. Calibration curve samples went into the sample preparation section. Calibration curves for AMO, AMA, and DIKETO in the liver, kidney, and urine were constructed using AMP at a concentration of 1 μg/g or μg/mL as an internal standard, the concentration ratios (x) between AMO, AMA, DIKETO, and AMP were used as the horizontal coordinates, and the peak area ratios (y) were used as the vertical coordinates. The standard curve was constructed by taking the analyte concentration (c) as the horizontal coordinate and the measured peak area (s) as the vertical coordinate for SBT in the liver, kidney, and urine and AMA, DIKETO, and SBT in feces. The correlation coefficients (r) were determined, and these values should all be ≥0.99.

#### 4.2.4. Recovery and Precision

Recovery and intra-day precision were estimated by analyzing three spiked concentrations (1 μg/mL or μg/g, 5 μg/mL or μg/g, and 10 μg/mL or μg/g), each of them with five replicates of each tissue. Interday precision was evaluated by repeating the procedure in three separate batches. The recovery rates of AMO, AMA, and DIKETO in the liver, kidney, and urine were calculated by the ratio of the peak area and the ratio of spiked concentration between AMO, AMA, DIKETO, and AMP. The recovery rates of SBT in the liver, kidney, and urine and AMA, DIKETO, and SBT in feces were calculated by the peak area and spiked concentration of AMA, DIKETO, and SBT.

### 4.3. Residual Elimination of Major AS Metabolites in Rats

#### 4.3.1. Experimental Design and Groups

Healthy male and female SD rats (SPF grade, SCXK (Jing) 2019-0010) weighing 180–220 g were used in this study. All the animals were reared under standardized conditions (a relative humidity of 60%, a temperature of 21 °C, and a 12 h light/dark cycle) and allowed free access to a standard diet and water. Animal experiments were conducted in strict agreement with protocols approved by the Institutional Animal Care and Use Committee of South China Agricultural University.

Among the 114 SPF-grade rats, half were male and half were female. The test rats were randomly divided into a blank control group and Groups A and B. There were 6 rats in the blank control group and 54 rats each in Groups A and B. Groups A and B were divided into 9 groups of 6 rats each. Group A was orally gavaged with 17.75 mg/kg b.w. (equivalent to 10 mg/kg b.w. of amoxicillin and 6.38 mg/kg b.w. of sulbactam) of the hybrid molecule of AS, Group B was orally gavaged with a mixture of 10 mg/kg b.w. of amoxicillin and 6.38 mg/kg b.w. of sulbactam, and the blank group was gavaged with saline. The rats fasted for 12 h before the test and 4 h after administration of the drug and were only able to access water freely.

#### 4.3.2. Sample Collection

The rats were weighed, and the drug was administered by gavage according to the rat’s body weight. The 114 rats were placed in metabolic cages, 3 to a cage. The rats were anesthetized at ether at 0.5 h, 1 h, 2 h, 4 h, 8 h, 12 h, 24 h, 48 h, or 72 h after drug administration, killed by spinal dislocation, and dissected. The livers and kidneys of the rats were removed, and urine and feces were collected. All samples were combined separately, homogenized (the feces were weighed in advance), and stored at −80 °C until analysis.

#### 4.3.3. Determination of Sample Concentrations

The rat samples were processed and analyzed by UPLC-MS/MS according to the method in “Section 4.2.1”. The sample concentration was determined three times. The peak area in the chromatogram produced by each analyte was recorded, and the concentrations of the main metabolites in each group of rat samples at different times were calculated using the linear equation.

#### 4.3.4. Data Analysis

The concentrations of the major metabolites of AS and the mixtures in the rat liver and kidney were log-transformed, and a time regression analysis was carried out. The regression equation was obtained as LnC = LnC0^−kt^ (C: concentration of the major metabolite at a time t, C0: initial concentration, and k: elimination rate constant) and used to calculate the elimination equation for the major metabolite in each tissue (C = C0e^−kt^) and the elimination half-life (t_1/2β_). *t*-tests were performed on the half-life parameters using SPSS 24.0 biostatistics software, and the t_1/2β_ values for AS and the mixture in the livers and kidneys of rats were compared. The urinary and fecal excretion rates were calculated as

$$\mathrm{Elimination}\;\mathrm{Rate}\;(\%)=\frac{m\times C}{M}$$

where m denotes the volume of urine in mL or the total quantity of feces in g; C denotes the concentration of the analyte in the sample, and M denotes the mass of the drug administered in mg.

## 5. Conclusions

In this study, a rapid, sensitive, and reliable method was developed and validated for the determination of amoxicillin, amoxicillinic acid, amoxicillin diketopiperazine, and sulbactam in rat liver, kidney, urine, and feces samples. The established method was used to investigate the residual elimination of the amoxicillin–sulbactam hybrid molecule (AS) in rats. The results indicated that the AS was rapidly absorbed internally and had a short time to peak, enabling rapid elimination by the organism. The primary route of excretion was through urine, with excretion rates of 60.61 ± 2.13% and 9.54 ± 0.26% via urine and feces, respectively. The residual elimination pattern of AS in rats investigated in this study provides a theoretical basis for the in-depth development and application of AS, as well as guidelines for the development of similar drugs.

## Figures and Tables

**Table 1:** LODs and LOQs of amoxicillin (AMO), amoxicillinoic acid (AMA), amoxicillin-diketopiperazine (DIKETO), and sulbactam (SBT) in five matrices.

Parameter	Analyte	Matrix			
		Liver	Kidneys	Urine	Feces
LOD (μg/mL or μg/g)	AMO	0.01	0.01	0.01	—
	AMA	0.01	0.01	0.01	0.025
	DIKETO	0.0025	0.0025	0.0025	0.01
	SBT	0.01	0.01	0.01	0.025
LOQ (μg/mL or μg/g)	AMO	0.025	0.025	0.025	—
	AMA	0.025	0.025	0.025	0.05
	DIKETO	0.01	0.01	0.01	0.025
	SBT	0.025	0.025	0.025	0.05

Note: “—” denotes not detected.

**Table 2:** Elimination parameters of major metabolites in the liver and kidney of rats in the AS and mixture groups.

Matrix	Metabolite	Group	Equation	Elimination Half-Life (h)
Liver	Total amoxicillin and its metabolites	AS group	C = 8.71e^−0.28t^	2.44 ± 0.05 *
		Mixture group	C = 9.35e^−0.31t^	2.32 ± 0.05
	Amoxicillin	AS group	C = 2.33e^−0.29t^	2.40 ± 0.06
		Mixture group	C = 2.16e^−0.30t^	2.30 ± 0.11
	Amoxicillinic acid	AS group	C = 7.84e^−0.38t^	1.82 ± 0.20 *
		Mixture group	C = 9.03e^−0.41t^	1.70 ± 0.11
	Sulbactam	AS group	C = 3.93e^−0.31t^	2.21 ± 0.30
		Mixture group	C = 3.45e^−0.31t^	2.17 ± 0.20
Kidney	Total amoxicillin and its metabolites	AS group	C = 3.36e^−0.26t^	2.74 ± 0.10 *
		Mixture group	C = 3.56e^−0.28t^	2.54 ± 0.04
	Amoxicillinic acid	AS group	C = 3.22e^−0.33t^	2.11 ± 0.08 **
		Mixture group	C = 3.89e^−0.38t^	1.84 ± 0.10
	Sulbactam	AS group	C = 2.95e^−0.30t^	2.28 ± 0.15
		Mixture group	C = 3.12e^−0.32t^	2.18 ± 0.08

Note: * denotes a significant correlation at the 0.05 level (2-tailed), ** denotes a significant correlation at the 0.01 level (2-tailed).

**Table 3:** Gradient elution program for AMO, AMA, DIKETO, and AMP used in ultra-performance liquid chromatography (UPLC).

Time (min)	Flow Rate (μL/min)	0.1% Formic Acid in Acetonitrile (%)	0.1% Formic Acid in Water (%)
0.0	350	10	90
0.5	350	10	85
1.0	350	90	10
3.5	350	90	10
3.8	350	10	90
5.0	350	10	90

**Table 4:** Mass spectral parameters of AMO, AMA, DIKETO, and AMP.

Analyte	Precursor Ions (*m*/*z*)	Product Ions (*m*/*z*)	Declustering Potential (V)	Collision Energy (eV)
AMO	366.0	114.1 *	49	27
		208.1	54	19
AMA	383.8	323.2 *	38	18
		367.1	32	15
DIKETO	365.7	160.3 *	53	27
		207.3	48	19
AMP	349.7	106.1 *	26	17
		192.1	30	18

Note: * Transition used for quantification. Abbreviations: AMO, amoxicillin; AMA, amoxicilloic acid; DIKETO, amoxicillin diketopiperazine; AMP, ampicillin.

**Table 5:** Gradient elution program used for ultra-performance liquid chromatography of SBT.

Time (min)	Flow Rate (μL/min)	0.1% Formic Acid in Acetonitrile (%)	0.1% Formic Acid in Water (%)
0.0	300	5	95
1.0	300	30	70
2.0	300	90	10
4.0	300	90	10
4.2	300	5	95
6.0	300	5	95

**Table 6:** Mass spectral parameters of SBT.

Analyte	Precursor Ions (*m*/*z*)	Product Ions (*m*/*z*)	Declustering Potential (V)	Collision Energy (eV)
Sulbactam	232.2	140.0 *	−35	−17
		96	−35	−17
		64	−35	−48

Note: * Transition used for quantification.

## Data Availability

Data are contained within the article.

## Appendix B

**Table A1:** Linear equation, correlation coefficient, and linearity range of amoxicillin in rat samples.

Matrix	Batch	Linearity Range (μg/mL or μg/g)	Linear Equation	Correlation Coefficient
Urine	1	0.025~10	y = 0.5629x − 0.0351	0.9994
	2	0.025~10	y = 0.4438x + 0.0378	0.9985
	3	0.025~10	y = 0.4947x − 0.0348	0.9996
Liver	1	0.025~10	y = 0.7677x − 0.0668	0.9991
	2	0.025~10	y = 0.6209x − 0.0507	0.9993
	3	0.025~10	y = 0.6152x + 0.0300	0.9948
Kidney	1	0.025~10	y = 0.6462x − 0.0194	0.9997
	2	0.025~10	y = 0.5698x + 0.0220	0.9994
	3	0.025~10	y = 0.5861x + 0.0335	0.9992

**Table A2:** Linear equation, correlation coefficient, and linearity range of amoxicilloic acid in rat samples.

Matrix	Batch	Linearity Range (μg/mL or μg/g)	Linear Equation	Correlation Coefficient
Urine	1	0.025~10	y = 0.1804x + 0.0009	0.9994
	2	0.025~10	y = 0.2103x + 0.0422	0.9991
	3	0.025~10	y = 0.1782x + 0.0163	0.9992
Liver	1	0.025~10	y = 0.2665x + 0.0334	0.9987
	2	0.025~10	y = 0.2204x + 0.0181	0.9994
	3	0.025~10	y = 0.2165x + 0.0097	0.9995
Kidney	1	0.025~10	y = 0.2541x + 0.0036	0.9992
	2	0.025~10	y = 0.1845x + 0.0147	0.9991
	3	0.025~10	y = 0.2306x − 0.0136	0.9996
Feces	1	0.05~10	s = 317,516c + 36,850	0.9985
	2	0.05~10	s = 329,082c + 40,839	0.9938
	3	0.05~10	s = 339,320c + 51,023	0.9929

**Table A3:** Linear equation, correlation coefficient, and linearity range of amoxicillin diketopiperazine in rat samples.

Matrix	Batch	Linearity Range (μg/mL or μg/g)	Linear Equation	Correlation Coefficient
Urine	1	0.001~10	y = 1.0708x − 0.0057	0.9993
	2	0.001~10	y = 0.8586x − 0.0583	0.9982
	3	0.001~10	y = 0.8617x + 0.0322	0.9975
Liver	1	0.001~10	y = 0.7537x − 0.0916	0.9992
	2	0.001~10	y = 0.7073x − 0.0755	0.9935
	3	0.001~10	y = 0.7404x − 0.0651	0.9994
Kidney	1	0.001~10	y = 0.6684x − 0.0611	0.9991
	2	0.001~10	y = 0.7204x − 0.0601	0.9995
	3	0.001~10	y = 0.7515x + 0.0029	0.9942
Feces	1	0.0025~10	s = 429,253c − 15,595	0.9992
	2	0.0025~10	s = 330,080c + 9065.5	0.9962
	3	0.0025~10	s = 343,413c + 16,205	0.9957

**Table A4:** Linear equation, correlation coefficient, and linearity range of sulbactam in rat samples.

Matrix	Batch	Linearity Range (μg/mL or μg/g)	Linear Equation	Correlation Coefficient
Urine	1	0.025~10	s = 125,656c + 5734.3	0.9988
	2	0.025~10	s = 142,231c + 4139.1	0.9992
	3	0.025~10	s = 128,916c + 9636.3	0.9976
Liver	1	0.025~10	s = 608,799c + 20,531	0.9993
	2	0.025~10	s = 525,809c −13,796	0.9995
	3	0.025~10	s = 501,645c + 16,491	0.9983
Kidney	1	0.025~10	s = 649,630c + 40,793	0.9963
	2	0.025~10	s = 532,714c + 15,408	0.9992
	3	0.025~10	s = 711,610c + 1353.9	0.9956
Feces	1	0.05~10	s = 610,688c − 44,116	0.9942
	2	0.05~10	s = 667,957c − 65,813	0.9994
	3	0.05~10	s = 755,649c + 13,200	0.9995

**Table A5:** Recovery and precision of amoxicillin in rat samples.

Matrix	Added Concentration (μg/mL or μg/g)	Recovery Rate ($\bar{X}$ ± S.D., %, *n* = 5)			Intra-Batch Precision (%, *n* = 5)			Inter-Batch Precision (%, *n* = 15)
		1	2	3	1	2	3	
Urine	1	97.65 ± 8.19	99.28 ± 5.56	103.87 ± 6.02	8.39	5.60	5.80	6.58
	5	103.39 ± 6.65	104.66 ± 7.43	106.94 ± 10.37	6.44	7.10	9.69	7.52
	10	101.25 ± 3.96	111.46 ± 7.84	108.56 ± 7.72	3.91	7.04	7.11	7.22
Liver	1	91.55 ± 1.26	96.89 ± 3.20	96.64 ± 2.58	1.37	3.30	2.67	3.61
	5	98.43 ± 2.51	105.49 ± 7.19	95.81 ± 5.35	2.55	6.82	5.58	6.54
	10	97.52 ± 7.97	99.58 ± 9.10	102.04 ± 4.22	8.17	9.14	4.14	7.13
Kidney	1	97.32 ± 2.64	99.70 ± 4.89	98.93 ± 3.29	2.72	4.90	3.32	3.53
	5	102.10 ± 2.91	99.27 ± 3.40	99.39 ± 5.51	2.85	3.42	5.54	4.02
	10	102.69 ± 8.51	105.32 ± 1.51	106.34 ± 2.34	8.28	1.43	2.20	4.81

**Table A6:** Recovery and precision of amoxicilloic acid in rat samples.

Matrix	Added Concentration (μg/mL or μg/g)	Recovery Rate ($\bar{X}$ ± S.D., %, *n* = 5)			Intra-Batch Precision (%, *n* = 5)			Inter-Batch Precision (%, *n* = 15)
		1	2	3	1	2	3	
Urine	1	89.72 ± 7.34	86.69 ± 4.21	80.56 ± 9.21	8.18	4.85	11.44	9.03
	5	90.48 ± 6.97	85.92 ± 7.94	83.17 ± 3.65	7.70	9.24	4.39	7.54
	10	91.04 ± 4.14	95.70 ± 5.00	94.34 ± 5.57	4.55	5.23	5.90	5.38
Liver	1	107.20 ± 2.82	109.71 ± 7.67	100.72 ± 2.61	2.63	6.99	2.60	6.43
	5	110.11 ± 4.79	110.45 ± 7.99	107.07 ± 10.76	4.35	7.23	10.05	6.77
	10	103.96 ± 9.75	105.83 ± 7.86	109.12 ± 8.92	9.38	7.43	8.17	8.01
Kidney	1	110.62 ± 5.50	99.35 ± 4.31	100.31 ± 3.25	4.97	4.34	3.24	6.62
	5	109.02 ± 3.72	106.40 ± 9.05	107.66 ± 9.94	3.41	8.50	9.23	7.00
	10	109.17 ± 8.12	110.04 ± 6.51	109.29 ± 8.16	7.44	5.92	7.47	6.47
Feces	1	81.84 ± 6.70	84.54 ± 6.28	81.28 ± 7.43	8.24	7.43	9.14	7.74
	5	87.86 ± 7.59	89.57 ± 5.55	81.32 ± 5.45	8.63	6.20	6.70	7.86
	10	85.14 ± 3.04	84.69 ± 3.97	88.32 ± 2.12	3.56	4.69	2.40	3.83

**Table A7:** Recovery and precision of amoxicillin diketopiperazine in rat samples.

Matrix	Added Concentration (μg/mL or μg/g)	Recovery Rate ($\bar{X}$ ± S.D., %, *n* = 5)			Intra-Batch Precision (%, *n* = 5)			Inter-Batch Precision (%, *n* = 15)
		1	2	3	1	2	3	
Urine	1	96.11 ± 7.16	94.65 ± 10.81	101.83 ± 9.66	7.45	11.42	9.49	9.58
	5	99.61 ± 5.37	105.82 ± 8.90	103.90 ± 8.66	5.39	8.42	8.34	7.34
	10	101.83 ± 5.92	110.34 ± 9.18	110.92 ± 6.68	5.82	8.32	6.02	7.45
Liver	1	103.52 ± 6.93	108.31 ± 9.88	97.44 ± 7.51	6.70	9.12	7.70	8.62
	5	104.94 ± 2.66	104.86 ± 9.06	94.50 ± 6.44	2.53	8.64	6.82	7.83
	10	103.11 ± 7.89	99.62 ± 9.34	105.79 ± 11.10	7.65	9.37	10.49	8.95
Kidney	1	101.38 ± 7.68	92.76 ± 6.99	91.59 ± 9.72	7.57	7.54	10.61	9.19
	5	104.07 ± 6.81	91.39 ± 6.11	98.31 ± 6.73	6.54	6.69	6.84	8.27
	10	102.62 ± 9.81	101.35 ± 9.57	105.69 ± 9.54	9.56	9.45	9.02	8.84
Feces	1	88.28 ± 8.00	86.09 ± 5.68	80.07 ± 6.10	9.06	6.60	7.62	8.30
	5	86.91 ± 8.27	84.95 ± 8.53	90.40 ± 7.10	9.52	10.04	7.86	9.50
	10	82.9 ± 3.42	91.16 ± 7.14	89.27 ± 3.25	4.12	7.83	3.64	5.41

**Table A8:** Recovery and precision of sulbactam in rat samples.

Matrix	Added Concentration (μg/mL or μg/g)	Recovery Rate ($\bar{X}$ ± S.D., %, *n* = 5)			Intra-Batch Precision (%, *n* = 5)			Inter-Batch Precision (%, *n* = 15)
		1	2	3	1	2	3	
Urine	1	96.93 ± 5.54	96.11 ± 3.06	97.62 ± 5.37	5.72	3.18	5.50	4.63
	5	98.24 ± 5.90	97.13 ± 4.28	95.05 ± 8.70	6.01	4.41	9.15	6.42
	10	96.39 ± 4.38	94.05 ± 3.48	94.78 ± 6.05	4.54	3.70	6.38	4.75
Liver	1	78.15 ± 8.94	84.63 ± 6.94	74.34 ± 6.84	11.44	8.20	9.20	10.53
	5	79.99 ± 7.11	80.70 ± 7.20	80.62 ± 6.79	8.89	8.92	8.43	8.11
	10	81.25 ± 3.42	84.14 ± 5.49	74.76 ± 8.97	4.21	6.52	12.00	9.22
Kidney	1	75.34 ± 5.64	78.19 ± 5.58	82.73 ± 5.39	7.48	7.13	6.51	7.64
	5	71.59 ± 6.78	79.43 ± 8.34	80.27 ± 6.65	9.48	10.50	8.29	10.22
	10	72.23 ± 5.48	75.43 ± 6.62	81.62 ± 4.05	7.59	8.77	4.96	8.49
Feces	1	80.41 ± 7.91	83.70 ± 5.58	80.12 ± 6.78	9.83	6.66	8.46	8.09
	5	76.98 ± 3.41	79.97 ± 4.88	82.02 ± 9.63	4.43	6.10	11.74	8.06
	10	84.44 ± 4.75	82.54 ± 4.76	83.15 ± 6.95	5.62	5.76	8.36	6.28

**Table A9:** Concentrations of major metabolites in the liver at different time points in the AS group.

Time (h)	Mean Drug Concentration (μg/g) ± S.D.				
	Amoxicillin	Amoxicilloic Acid	Amoxicillin Diketopiperazine	Total Amoxicillin	Sulbactam
0.5	2.503 ± 0.128	2.488 ± 0.090	0.636 ± 0.029	5.627 ± 0.325	2.892 ± 0.170
1	3.516 ± 0.223	3.037 ± 0.301	0.469 ± 0.020	7.022 ± 0.491	3.499 ± 0.154
2	1.882 ± 0.131	2.452 ± 0.213	0.298 ± 0.018	4.631 ± 0.392	2.256 ± 0.189
4	0.630 ± 0.041	2.052 ± 0.165	0.223 ± 0.044	2.905 ± 0.166	1.141 ± 0.132
8	0.127 ± 0.017	0.692 ± 0.035	0.161 ± 0.015	0.980 ± 0.039	0.346 ± 0.051
12	0.107 ± 0.015	0.053 ± 0.016	0.112 ± 0.021	0.273 ± 0.043	0.098 ± 0.040
24	ND	ND	ND	ND	0.038 ± 0.008
48	ND	ND	ND	ND	ND
Note: Total amoxicillin is the total concentration of amoxicillin, amoxicillinic acid, and amoxicillin diketopiperazine. “ND” means not detected.					

**Table A10:** Concentrations of major metabolites in the liver at different time points in the mixture group.

Time (h)	Mean Drug Concentration (μg/g) ± S.D.				
	Amoxicillin	Amoxicilloic Acid	Amoxicillin Diketopiperazine	Total Amoxicillin	Sulbactam
0.5	3.013 ± 0.171	2.635 ± 0.149	0.620 ± 0.051	6.268 ± 0.369	3.569 ± 0.193
1	3.432 ± 0.126	3.608 ± 0.158	0.479 ± 0.035	7.520 ± 0.386	3.895 ± 0.272
2	1.693 ± 0.117	2.908 ± 0.104	0.281 ± 0.019	4.882 ± 0.343	2.093 ± 0.142
4	0.542 ± 0.014	1.827 ± 0.164	0.205 ± 0.018	2.575 ± 0.182	0.100 ± 0.068
8	0.110 ± 0.002	0.719 ± 0.069	0.143 ± 0.016	0.972 ± 0.075	0.331 ± 0.014
12	0.083 ± 0.018	0.044 ± 0.007	0.081 ± 0.013	0.208 ± 0.015	0.080 ± 0.021
24	ND	ND	ND	ND	0.036 ± 0.003
48	ND	ND	ND	ND	ND
Note: Total amoxicillin is the total concentration of amoxicillin, amoxicillinic acid, and amoxicillin diketopiperazine. “ND” means not detected.					

**Table A11:** Concentrations of major metabolites in the kidney at different time points in the AS group.

Time (h)	Mean Drug Concentration(μg/g) ± S.D.				
	Amoxicillin	Amoxicilloic Acid	Amoxicillin Diketopiperazine	Total Amoxicillin	Sulbactam
0.5	0.480 ± 0.013	2.115 ± 0.144	0.297 ± 0.019	2.840 ± 0.175	1.646 ± 0.056
1	0.277 ± 0.010	2.607 ± 0.257	0.249 ± 0.024	3.187 ± 0.151	1.919 ± 0.150
2	0.234 ± 0.007	1.904 ± 0.197	0.192 ± 0.016	2.436 ± 0.117	1.386 ± 0.117
4	0.163 ± 0.002	0.726 ± 0.051	0.135 ± 0.012	1.014 ± 0.047	0.963 ± 0.065
8	0.075 ± 0.002	0.241 ± 0.018	0.107 ± 0.009	0.403 ± 0.017	0.310 ± 0.018
12	ND	0.064 ± 0.006	0.092 ± 0.012	0.176 ± 0.023	0.068 ± 0.012
24	ND	ND	ND	ND	ND
Note: Total amoxicillin is the total concentration of amoxicillin, amoxicillinic acid, and amoxicillin diketopiperazine. “ND” means not detected.					

**Table A12:** Concentrations of major metabolites in the kidney at different time points in the mixture group.

Time (h)	Mean Drug Concentration (μg/g) ± S.D.				
	Amoxicillin	Amoxicilloic Acid	Amoxicillin Diketopiperazine	Total Amoxicillin	Sulbactam
0.5	0.467 ± 0.013	2.380 ± 0.117	0.309 ± 0.064	3.155 ± 0.125	1.846 ± 0.102
1	0.251 ± 0.032	2.987 ± 0.122	0.259 ± 0.012	3.497 ± 0.149	2.142 ± 0.476
2	0.162 ± 0.003	2.026 ± 0.112	0.178 ± 0.025	2.265 ± 0.153	1.457 ± 0.290
4	0.121 ± 0.005	0.805 ± 0.063	0.135 ± 0.012	1.061 ± 0.090	0.991 ± 0.135
8	0.069 ± 0.003	0.171 ± 0.019	0.100 ± 0.011	0.340 ± 0.015	0.266 ± 0.017
12	ND	0.046 ± 0.002	0.099 ± 0.005	0.145 ± 0.012	0.065 ± 0.005
24	ND	ND	ND	ND	ND
Note: Total amoxicillin is the total concentration of amoxicillin, amoxicillinic acid, and amoxicillin diketopiperazine. “ND” means not detected.					

**Table A13:** Table of urinary excretion of major metabolites after administration in the AS group.

Time (h)	Excretion Rate (%) ± S.D.				
	Amoxicillin	Amoxicilloic Acid	Amoxicillin Diketopiperazine	Sulbactam	Total
0.5	ND	ND	ND	ND	ND
1	0.97 ± 0.08	2.52 ± 0.15	ND	1.47 ± 0.15	4.97 ± 0.35
2	2.11 ± 0.12	4.11 ± 0.34	1.01 ± 0.09	2.73 ± 0.13	9.95 ± 0.75
4	4.13 ± 0.21	6.88 ± 0.50	1.72 ± 0.12	5.63 ± 0.36	18.36 ± 0.81
8	6.36 ± 0.46	12.59 ± 1.01	3.08 ± 0.23	12.31 ± 0.81	34.36 ± 1.25
12	8.42 ± 0.34	16.68 ± 1.28	4.69 ± 0.35	15.01 ± 1.05	44.78 ± 1.66
24	10.12 ± 0.62	20.25 ± 1.10	5.50 ± 0.40	16.44 ± 1.03	52.30 ± 1.84
48	10.83 ± 0.72	24.36 ± 1.41	6.99 ± 0.50	17.44 ± 1.42	59.62 ± 2.50
72	10.98 ± 0.65	24.26 ± 1.36	7.15 ± 0.42	18.22 ± 1.30	60.61 ± 2.13
Note: Total is the total excretion rate of amoxicillin, amoxicillinic acid, amoxicillin diketopiperazine, and sulbactam. “ND” means not detected.					

**Table A14:** Table of urinary excretion of major metabolites after administration in the mixture group.

Time (h)	Excretion Rate (%) ± S.D.				
	Amoxicillin	Amoxicilloic Acid	Amoxicillin Diketopiperazine	Sulbactam	Total
0.5	ND	ND	ND	ND	ND
1	1.20 ± 0.06	2.55 ± 0.13	ND	1.54 ± 0.18	5.28 ± 0.30
2	2.85 ± 0.17	3.74 ± 0.12	0.97 ± 0.03	2.84 ± 0.11	10.41 ± 0.76
4	5.09 ± 0.46	6.60 ± 0.44	1.80 ± 0.11	6.02 ± 0.32	19.50 ± 0.73
8	7.01 ± 0.41	12.80 ± 0.72	3.23 ± 0.24	10.63 ± 0.76	33.67 ± 1.25
12	9.35 ± 0.53	17.14 ± 0.68	4.35 ± 0.19	14.97 ± 1.19	45.81 ± 1.46
24	11.11 ± 0.87	21.87 ± 1.12	5.83 ± 0.33	16.81 ± 1.13	55.63 ± 1.14
48	11.87 ± 0.62	22.52 ± 1.21	8.05 ± 0.47	18.11 ± 1.04	60.55 ± 1.55
72	11.68 ± 0.76	23.52 ± 1.04	8.40 ± 0.62	19.02 ± 1.38	62.62 ± 1.73
Note: Total is the total excretion rate of amoxicillin, amoxicillinic acid, amoxicillin diketopiperazine, and sulbactam. “ND” means not detected.					

**Table A15:** Excretion table of major metabolites in feces after administration in the AS group.

Time (h)	Excretion Rate (%) ± S.D.			
	Amoxicilloic Acid	Amoxicillin Diketopiperazine	Sulbactam	Total
0.5	ND	ND	ND	ND
1	ND	ND	ND	ND
2	0.01	0.01	0.02	0.04
4	0.02	0.01	0.02	0.05
8	0.02	0.02	0.02	0.06
12	0.12 ± 0.01	0.09	0.23 ± 0.04	0.44 ± 0.02
24	0.95 ± 0.07	1.36 ± 0.08	2.29 ± 0.13	3.61 ± 0.11
48	2.62 ± 0.11	1.85 ± 0.15	4.39 ± 0.14	8.85 ± 0.12
72	2.85 ± 0.12	2.14 ± 0.16	4.35 ± 0.11	9.54 ± 0.26
Note: Total is the total excretion rate of amoxicillin, amoxicillinic acid, amoxicillin diketopiperazine, and sulbactam. “ND” means not detected.				

**Table A16:** Table of excretion of major metabolites in feces after administration of the mixture group.

Time (h)	Excretion rate (%) ± S.D.			
	Amoxicilloic Acid	Amoxicillin Diketopiperazine	Sulbactam	Total
0.5	ND	ND	ND	ND
1	ND	ND	ND	ND
2	0.03	0.01	0.03	0.08
4	0.04	0.02	0.06	0.12
8	0.08	0.05	0.17 ± 0.01	0.29
12	1.14 ± 0.08	1.15 ± 0.07	1.46 ± 0.07	3.76 ± 0.16
24	2.12 ± 0.12	1.46 ± 0.08	3.88 ± 0.11	7.46 ± 0.11
48	3.08 ± 0.11	2.50 ± 0.14	5.09 ± 0.14	10.67 ± 0.25
72	2.87 ± 0.17	2.42 ± 0.11	5.32 ± 0.15	10.60 ± 0.24
Note: Total is the total excretion rate of amoxicillin, amoxicillinic acid, amoxicillin diketopiperazine, and sulbactam. “ND” means not detected.				
